# Psychological and Behavioral Predictors of Postpartum Lumbopelvic Pain: A Multivariate Analysis

**DOI:** 10.3390/medicina61101869

**Published:** 2025-10-17

**Authors:** Ignacio Jiménez-de-Ory, Angelika Mazur, Ángel Oliva-Pascual-Vaca, María Benito-de-Pedro, Tomás Fernández-Rodríguez, Elena Sonsoles Rodríguez-López

**Affiliations:** 1 Physiotherapy and Health Research Group (FYSA), Department of Physiotherapy, Faculty of Health Sciences—HM Hospitals, University Camilo José Cela, Villanueva de la Cañada, 28692 Madrid, Spain; ignacio.jimenezd@ucjc.edu (I.J.-d.-O.); angelika.mazur@alumno.ucjc.edu (A.M.); mbenito@ucjc.edu (M.B.-d.-P.); tfernandez@ucjc.edu (T.F.-R.); 2 HM Hospitals Health Research Institute, 28015 Madrid, Spain; 3 Instituto de Biomedicina de Sevilla (IBiS), Departamento de Fisioterapia, Universidad de Sevilla, 41013 Seville, Spain; angeloliva@us.es

**Keywords:** lumbopelvic pain, postpartum, postpartum depression, sleep quality, kinesiophobia

## Abstract

*Background and Objectives:* Postpartum lumbopelvic pain (PLPP) is a common condition that negatively affects many women’s quality of life. We aimed to analyze the influence of emotional well-being, kinesiophobia, and sleep quality as predictors of PLPP during the first year postpartum. *Materials and Methods*: A cross-sectional study was conducted with 192 women in their first year postpartum. Validated questionnaires were administered to evaluate pain (Oswestry Disability Index, ODI), postpartum depression (PPD) (Edinburgh Postnatal Depression Scale, EPDS), sleep quality (Pittsburgh Sleep Quality Index, PSQI), and kinesiophobia (Tampa Scale of Kinesiophobia, TSK-11). Bivariate correlations and binary logistic regression were performed to identify predictors of PLPP. *Results*: Overall, 42.2% of participants reported lumbopelvic pain. The prevalence of postpartum depressive symptoms was 59.9%, and kinesiophobia was present in 30.7% of women with pain. Both PPD and kinesiophobia were significantly associated with the presence of PLPP (*p* < 0.001). In the multivariate model, depression was the main predictor (*OR* = 8.1), followed by kinesiophobia (*OR* = 3.6). Sleep quality was not an independent predictor but may be related to PLPP through indirect mechanisms. No significant associations were found with sociodemographic, obstetric, or lifestyle variables. *Conclusions*: PPD and kinesiophobia are key factors in the occurrence of PLPP, while sleep quality may act as a mediating variable. These findings highlight the need for postnatal interventions addressing emotional health and fear of movement to improve the prevention and management of lumbopelvic pain in this population.

## 1. Introduction

Lumbopelvic pain (LPP) is a common complaint during pregnancy, affecting an estimated 50% to 70% of women [1,2]. Recovery from pregnancy-related LPP is common; a prospective cohort study reported that approximately 78% of women experience spontaneous improvement within six weeks of delivery [3]. However, recovery rates vary across studies depending on methodology and follow-up duration. Nearly one-third of participants still report symptoms at three months [2], and recent data indicate that LPP remains highly prevalent during the first postpartum year, affecting up to 66% of women [4]. Notably, one in ten women develops pelvic girdle pain (PGP) with severe, long-lasting effects, impacting daily functioning, family life, and socioeconomic stability up to 11 years after childbirth [5]. This condition, commonly referred to as postpartum lumbopelvic pain (PLPP), may present as low back pain, PGP, or both. Predictive models have identified biomechanical, physiological, social, economic, and obstetric factors as significant contributors to symptom persistence [6,7,8]. Although several physical and obstetric risk factors have been identified for PLPP, such as pre-existing low back pain during pregnancy, parity, or type of delivery, these factors alone do not fully explain the persistence of pain after childbirth [6,7,8].

Beyond physiological and biomechanical mechanisms, maternal psychological well-being is a critical factor in understanding PLPP [7,9]. Postpartum depression (PPD), poor sleep quality, and kinesiophobia not only influence pain perception but also hinder recovery and promote chronicity [10]. Emotional stress, sleep deprivation, and fear of movement can exacerbate physical discomfort during the first year postpartum. These interrelated factors underscore the need for a biopsychosocial framework in both the study and treatment of this condition [11]. Despite its high prevalence and significant impact, PLPP is often underestimated and under-recognized. Many women perceive this pain as an unavoidable part of motherhood, discouraging them from seeking medical care [4]. Such misconceptions compromise the quality of life and delay preventive and therapeutic interventions.

Emerging evidence suggests that a multidimensional approach—encompassing risk factor identification, tailored exercise programs, and psychosocial support—can effectively prevent and manage PLPP. These interventions may improve the well-being of affected women, reduce the long-term healthcare costs associated with chronic complications, and ultimately enhance maternal health outcomes [12].

This study aimed to examine the roles of emotional well-being, kinesiophobia, and sleep quality as protective or risk factors for PLPP during the first year postpartum using a multivariate approach. To our knowledge, it is the first to jointly evaluate these factors within a biopsychosocial model, offering insights that extend beyond prior studies that assessed them individually.

## 2. Materials and Methods

### 2.1. Study Design

This cross-sectional, descriptive study included women in their first year postpartum with the aim of evaluating the relationship between PLPP and sociodemographic, obstetric, and musculoskeletal factors. The study was approved by the Ethics Committee of the University Camilo José Cela (code 21_24_CLxP, 29 January 2025; Madrid, Spain). All participants received detailed information about the study and provided written informed consent before participating.

### 2.2. Participants and Inclusion and Exclusion Criteria

Postpartum women were recruited from pelvic floor physiotherapy clinics and obstetrics centers, and through peer recommendation among postpartum mothers. The sample size was calculated using G*Power software version 3.1.9.6 (Kiel University, Kiel, Germany). A two-tailed test with an effect size (d) of 0.5, an α error probability of 0.05, and a statistical power of 0.99 yielded a minimum required sample size of 108 postpartum women (approximately 46 with PLPP and 62 without). For the multivariate logistic regression analysis, the minimum sample size was estimated using Peduzzi’s formula [13], considering three main predictors (postpartum depression, kinesiophobia, and sleep quality) and a 42% prevalence of PLPP. This calculation indicated that at least 172 participants were required to ensure adequate power (≥10 events per variable). The final sample of 192 women, therefore, exceeded the required size for both the bivariate and multivariate analyses.

Eligibility criteria were as follows: (1) women within 12 months postpartum; (2) age ≥ 18 years; and (3) provided written informed consent to participate. Exclusion criteria were as follows: (1) pre-existing musculoskeletal disorders diagnosed before pregnancy; (2) neurological or rheumatological diseases affecting pain perception; (3) age < 18 years; (4) severe pregnancy or delivery complications; (5) inability to understand the questionnaire in Spanish; (6) history of pelvic (e.g., hysterectomy, cesarean section, pelvic floor repair), lumbar (e.g., discectomy, spinal fusion, laminectomy), and major abdominal surgeries (e.g., laparotomy, hernia repair) performed within the 12 months prior to participation; and (7) severe psychiatric disorder diagnosed prior to pregnancy (e.g., schizophrenia, bipolar disorder) that could affect pain perception or response accuracy [14].

### 2.3. Instruments and Data Collection

Data were collected between February and April 2025 using an anonymous online questionnaire hosted on Survey Monkey^®^ (2025) version 4.5.8 (San Mateo, CA, USA). Participants were invited to complete the questionnaire, and their information was automatically processed and stored on a password-protected server. The principal investigator managed the data in compliance with current data protection regulations. No compensation was provided.

The online questionnaire was previously evaluated to ensure usability and performance. Participants were instructed to review their responses before submission. The questionnaire consisted of seven sections: (1) anthropometric data and employment status; (2) medical history and habits; (3) obstetric data; (4) Oswestry Disability Index (ODI); (5) Pittsburgh Sleep Quality Index (PSQI); (6) Edinburgh Postnatal Depression Scale (EPDS); and (7) Tampa Scale of Kinesiophobia (TSK-11).

### 2.4. Study Variables

Maternal variables included age (years), weight (kg), height (cm), and employment status. Lifestyle factors, such as smoking, alcohol consumption, and level of physical activity (walking or sports practice, expressed in hours per day and weekly frequency), were also collected. Medical history included the presence of mental disorders. Obstetric data included date of last delivery, gestational age, type of delivery (vaginal, cesarean, or instrumental), and presence of episiotomy, tears, or postpartum hemorrhage. Additional information on previous pregnancies was recorded, including parity (primiparous or multiparous) and type of delivery in previous gestations. Neonatal data included birth weight (kg) and length (cm). Finally, the questionnaire included a question on whether the mother had experienced pain in the lumbar or pelvic area since childbirth and when the pain started (before or after pregnancy).

Lumbopelvic-pain-related disability was assessed using the ODI, a validated and widely used measure of functional limitation in individuals with low back pain [15]. The ODI consists of 10 self-reported items, each scored on a scale of 0 to 5. No clinical examination or medical diagnosis was performed to confirm pain status. Participants without lumbopelvic pain reported no functional limitations, resulting in an ODI score of 0. PLPP classification was based on participants’ self-reported perception, with prevalence determined based on affirmative responses to the item on pain since childbirth.

Sleep quality was assessed using the PSQI, which measures sleep quality and patterns over the previous month. This self-reported questionnaire includes 19 items assessing seven components, including sleep latency, duration, efficiency, and disturbances [16]. Total scores range from 0 to 21, with higher values indicating poorer sleep quality.

Maternal psychological status was assessed using the EPDS, a screening tool for depressive symptoms in pregnant and postpartum women [17]. The score for this 10-item self-reported questionnaire ranges from 0 to 30, with higher values indicating a greater risk of PPD. It has been validated in multiple languages, including Spanish [18], and it is widely used in clinical and research settings.

Fear of movement was assessed using the Spanish version of the TSK-11, a specialized tool for measuring kinesiophobia in individuals with musculoskeletal pain [19]. This 11-item short form has demonstrated good reliability and validity.

### 2.5. Statistical Analysis

Data analysis was performed using IBM Corp.’s (2018) SPSS Statistics for Windows Version 26.0 [computer software] (Armonk, NY, USA). Descriptive and inferential statistical techniques were applied. For descriptive analysis, measures of central tendency and dispersion (mean and standard deviation) were calculated for quantitative variables and frequencies (percentages) for qualitative variables. The distribution of quantitative variables was normal according to the Shapiro–Wilk test. To analyze differences between groups with and without PLPP or depression, Student’s t-test for independent samples was applied.

Bivariate analysis was performed using the Chi-square test to identify associations between PLPP in the first year postpartum (assessed through the ODI and psychological and functional variables: sleep quality (PSQI), PPD (EPDS), and kinesiophobia (TSK-11)). Pearson’s correlation was used to analyze associations between functional disability due to low back pain and variables related to psychological, sleep, and personal factors. The strength of the correlation was interpreted according to the following scale: |*r*| = 0.00–0.19 (very weak), 0.20–0.39 (weak), 0.40–0.59 (moderate), 0.60–0.79 (strong), and 0.80–1.00 (very strong) [20].

Binary logistic regression analysis was conducted to evaluate the contribution of depression (EPDS), kinesiophobia, and PSQI scores to the presence of PLPP. The model included these three independent variables and a constant term, allowing for estimation of adjusted odds ratios (ORs) for each predictor. Categorical variables were dummy-coded, and multicollinearity was evaluated using the variance inflation factor (VIF), with all values below 2, indicating no collinearity problems. A statistical significance level of *p* < 0.05 was applied in all analyses.

## 3. Results

This study included 192 women in their first year postpartum (mean age 35.6 ± 4.8 years; mean BMI 23.5 ± 4.5 kg/m^2^). Most had a university education (83.9%) and were employed (55.9%). Sociodemographic, lifestyle, and obstetric characteristics are summarized in Table 1.

**Figure 1:**
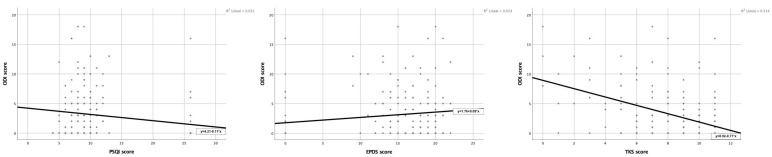
Scatter plots of the bivariate association of PLPP with sleep quality (**left**), postpartum depression (**center**), and kinesiophobia (**right**).

Participants reported mean scores of 2.99 (4.12) on the ODI, 11.54 (6.82) on the PSQI, 13.62 (7.14) on the EPDS, and 8.42 (2.70) on the TSK-11. These measures allowed for assessments of functional disability, sleep quality, depressive symptoms, and fear of movement, supporting the analysis of clinical associations in the first year postpartum, as presented below (bivariate associations) (Figure 1).

*PLPP and sleep quality.* PLPP was significantly associated with impaired sleep quality, as 76.2% of women with pain reported sleep disturbances compared with 63.7% without pain (χ^2^ = 5.06; *p* = 0.007). No women with pain reported good sleep, whereas 2.2% without pain did. A weak negative correlation was also found between PSQI and ODI (r = −0.175; *p* = 0.015).

*PLPP and PPD.* Women with PLPP reported more depressive symptoms (91.1% vs. 67.0% without pain (χ^2^ = 17.1; *p* < 0.001)). OR analysis showed that women with PLPP had five times greater odds of depressive symptoms (OR = 5.03). ODI and EPDS showed a weak but positive correlation (r = 0.156; *p* = 0.031).

*PLPP and kinesiophobia.* PLPP was also associated with kinesiophobia (30.7% vs. 8.8% without pain (χ^2^ = 14.2; *p* < 0.001; OR = 4.60)). A moderate negative correlation was found between ODI and TSK-11 (r = −0.463; *p* < 0.001).

*PPD and sleep quality.* The strongest association observed was between depressive symptoms and poor sleep quality (χ^2^ = 89.4; *p* < 0.001). Among depressed women, 85.6% reported poor sleep compared with 10.3% of non-depressed women. EPDS and PSQI showed a strong negative correlation (r = −0.860; *p* < 0.001).

*PPD and kinesiophobia.* Women with depressive symptoms were almost six times more likely to report kinesiophobia (χ^2^ = 6.97; *p* = 0.007; OR = 5.90). EPDS and TSK-11 were moderately correlated (r = 0.338; *p* < 0.001).

*PPD and neonatal weight.* A weak negative correlation was observed between EPDS and neonatal birth weight (r = −0.181; *p* = 0.043).

In the adjusted model (multivariate logistic regression), postpartum depression was the main predictor of PLPP (B = 2.09; SE = 0.62; Wald = 11.49; *p* = 0.0007; OR = 8.09). Kinesiophobia was also significant (B = 1.29; SE = 0.45; Wald = 8.37; *p* = 0.0038; OR = 3.6). Sleep quality was not an independent predictor (B = −0.83; SE = 0.54; Wald = 2.34; *p* = 0.126; OR = 0.44). The constant term was significant (B = −1.21; SE = 0.39; Wald = 9.75; *p* = 0.0018; OR = 0.30).

Overall, bivariate analyses showed that PLPP was associated with depressive symptoms, kinesiophobia, and sleep disturbances. In the multivariate model, depression and kinesiophobia remained independent predictors, whereas sleep quality lost significance.

## 4. Discussion

The results of this study confirm that maternal psychological well-being during the first year postpartum is a key factor in the onset of PLPP. In our sample of 192 women, 42.2% reported PLPP after childbirth, which is consistent with previous reports ranging from 25% to 45% during the first postnatal year [21,22,23]. Despite its high prevalence, pain at this stage remains under-recognized and often overlooked in healthcare [4,10,24].

One of the most notable findings was the high prevalence of PPD symptoms, reaching 59.9%. This rate exceeds the national rate in Spain, where recent longitudinal studies have reported prevalence rates of postpartum depressive symptoms between 25% and 30% during the first year after delivery [25]. This discrepancy may reflect sampling bias, reliance on self-reported tools, or sociocultural factors influencing help-seeking and symptom reporting [26]. Among women with pain, 91.1% presented with PPD, compared to 67% in the pain-free group, indicating a strong and significant association. Similar findings were reported by Madzivhandila et al. [9], who found that women with persistent postpartum pain were more likely to suffer from depression, which in turn negatively affected functional recovery [9].

This association may be explained through several mechanisms. Depression can amplify pain through alterations in neuroendocrine pathways involved in nociceptive perception, such as the hypothalamic–pituitary–adrenal axis [27]. Conversely, chronic pain may exacerbate depressive symptoms by affecting functionality, rest, self-esteem, and the maternal role [28].

In our relative risk analysis (OR = 5.027), the presence of pain significantly increased the likelihood of developing depressive symptoms, raising the risk fivefold. This result supports Long G et al. [29], who reported that PPD was three times more prevalent in women with PLPP than in those without [29]. Anxiety and depression may facilitate pain nociception, exacerbate symptoms, while pain itself can perpetuate anxiety and depression [5]. We also observed a positive correlation between ODI and EPDS (r = 0.156; *p* = 0.031), suggesting an interaction between physical and emotional distress. This reinforces the work of Gómez-Pérez L et al. [30], who found a bidirectional link between physical symptoms and PPD [30]. Due to the cross-sectional nature of our study, causal inferences cannot be established. The observed association between PLPP and psychological distress is likely reciprocal, reflecting complex neurobiological and cognitive–emotional interactions that mutually reinforce pain perception and affective symptoms. Kinesiophobia, defined as fear of movement due to pain or risk of injury, was also significantly related to PLPP [10]. In the pain group, 30.7% of women reported kinesiophobia compared with 8.8% of those without pain. This difference aligns with the findings of Lundberg et al. [31], who concluded that fear of movement contributes to physical disability in women with low back pain [31]. Avoidance behaviors stemming from fear may lead to muscle disuse, loss of mobility, and progressive worsening of pain, creating a vicious cycle of fear, inactivity, and dysfunction. An inverse association was observed between pain severity and kinesiophobia. Similar trends have been reported in chronic pain and postpartum populations, suggesting that habituation or acceptance-based coping may lessen fear of movement despite persistent pain [32,33].

Our results also showed that sleep quality was impaired in a considerable proportion of women with PLPP. Specifically, 76.2% of women with pain reported sleep disturbances compared with 63.7% in the pain-free group. Although this difference was statistically significant in the bivariate analysis, sleep quality was not an independent predictor in the multivariate model. This finding is consistent with the work of Okun et al. [34], who indicated that postpartum sleep disturbances are influenced by depressive symptoms and anxiety [34,35]. In our study, a strong negative correlation was found between sleep and depression scores, suggesting that sleep may act more as a mediating variable than a direct cause of pain.

Another finding of interest was the correlation between maternal depressive symptoms and neonatal weight, which showed a slight but statistically significant trend toward lower birth weight among infants of mothers with PPD. Although the effect size was small, this association is consistent with previous research linking maternal depression to suboptimal birth outcomes. Nasreen et al. [36] reported that depressive symptoms during pregnancy were associated with reduced birth weight after controlling for confounders. Jarde et al. [37] demonstrated in a meta-analysis an increased risk of low birth weight in infants of women with untreated antenatal depression. Other authors [38,39] also support this link between perinatal depression and adverse birth outcomes, including low birth weight and prematurity. These findings suggest a relationship between maternal emotional health and neonatal growth, although causality cannot be inferred from existing data.

Contrary to expectations, variables like maternal age, BMI, education level, type of delivery, and presence of obstetric complications were not found to be significantly associated with PLPP. In our sample, 83.9% of participants had a university education, but this was not a protective factor against pain. This finding is consistent with the work of Elden et al. [5], who reported that education level, in the absence of specific interventions, does not by itself reduce the incidence of musculoskeletal pain [5]. Moreover, some studies suggest that women with higher education levels may have greater self-awareness and a greater tendency to report emotional symptoms [40].

Our results support the biopsychosocial conceptualization of PLPP, as both depressive symptoms and kinesiophobia were independently associated with PLPP. This reinforces the idea that postpartum pain cannot be explained solely by physical or obstetric factors, highlighting the need for multidisciplinary assessment and management.

Systematic mental health screening, interventions addressing fear of movement, and promotion of guided physical activity should be considered essential components of postpartum care. Combined interventions, including education, exercise, and psychological support, have shown effectiveness in reducing musculoskeletal pain and improving maternal well-being [41,42].

Finally, it is important to remember that maternal physical and emotional health directly affects the quality of life of the mother–child dyad. The presence of PPD and persistent pain not only impacts maternal functionality but may also compromise bonding, breastfeeding, rest, and the baby’s emotional development [43]. Therefore, a comprehensive approach to PLPP should also include prevention, psycho-emotional support, and women-centered physiotherapy as key strategies for overall well-being postpartum [44,45,46].

### Limitations of the Study and Future Research Directions

Its cross-sectional design precludes causal inference, and the use of self-reported measures may introduce bias, particularly in the identification of PLPP without clinical confirmation and the assessment of sleep and pain intensity. The correlation observed between sleep quality and depressive symptoms should be interpreted with caution, as the study’s cross-sectional design does not permit causal conclusions. Future research should adopt longitudinal approaches, incorporate precise clinical evaluations, and consider multidisciplinary interventions integrating sleep hygiene education, emotional management, and guided exercise to address PLPP more effectively.

## 5. Conclusions

Postpartum depression and kinesiophobia emerged as significant predictors of postpartum lumbopelvic pain, while sleep quality did not show an independent association in the multivariate model. These findings underscore the importance of addressing emotional well-being and fear of movement within a biopsychosocial framework when evaluating postpartum women. Systematic screening and supportive interventions targeting these factors may help prevent or reduce the persistence of pain. Further longitudinal studies are warranted to clarify the directionality of these associations and to inform more targeted preventive and therapeutic strategies.

## Figures and Tables

**Table 1:** Anthropometric, educational, lifestyle, and obstetric characteristics of the mothers, together with neonatal data, assessed using the ODI, PSQI, EPDS, and TSK scores, according to the presence or absence of PLPP and PPD (n = 192).

	Total Sample (*n =* 192)		PLPP					PPD				
			Present (*n =* 81)		Absent (*n =* 62)		*p*-Value	Present (*n =* 115)		Absent (*n =* 28)		*p*-Value
	Mean	SD	Mean	SD	Mean	SD		Mean	SD	Mean	SD	
**Age (years)**	35.61	4.77	35.37	4.77	35.92	4.78	0.497 ^a^	35.71	4.98	35.18	3.82	0.597 ^a^
**BMI (kg/m^2^)**	23.50	4.45	23.28	3.91	23.78	5.08	0.508 ^a^	23.34	4.01	24.15	5.96	0.386 ^a^
**Maternal education level**												
Primary (n.%)	1	0.7%	0	0	1	1.6%	0.151 ^b^	0	0	1	3.6%	0.598 ^b^
Secondary school (n.%)	2	1.4%	0	0	2	3.2%		2	1.7%	0	0	
High school (n.%)	3	2.1%	2	2.5%	1	1.6%		3	2.6%	0	0	
Vocational training (n.%)	17	11.9%	11	13.6%	6	9.6%		14	12.2%	3	10.7%	
University (n.%)	120	83.9%	68	84.0%	52	83.8%		96	83.5%	24	85.7%	
**Maternal employment status**												
On maternity leave	48	33.6%	28	34.6%	20	32.26%	0.095 ^b^	42	36.5%	6	21.4%	0.799 ^b^
Unemployed	8	5.6%	3	3.7%	5	8%		6	5.2%	2	7.1%	
Housewife	1	0.7%	0	0	1	1.6%		1	0.9%	0	0	
Student	3	2.1%	3	3.7%	0	0		3	2.6%	0	0	
Student and working	3	2.1%	3	3.7%	0	0		3	2.6%	0	0	
Currently working	80	55.9%	44	54.3%	36	58%		60	52.2%	20	71.4%	
**Lifestyle**												
Smoke (yes (n.%))	6	4.2%	3	3.7%	3	4.8%	0.208 ^b^	4	3.5%	2	7.1%	0.612 ^b^
Alcohol (yes (n.%))	84	58.7%	45	55.6%	39	62.9%	0.197 ^b^	66	57.4%	18	64.3%	0.734 ^b^
Walking												
Hours/day	1.32	1.11	1.26	0.99	1.44	1.42	0.384 ^a^	1.34	1.16	1.35	1.38	0.989 ^a^
Days/week	3.93	2.58	3.75	2.60	4.12	2.41	0.401 ^a^	3.87	2.48	4.13	2.67	0.650 ^a^
Sports practice												
Hours/day	0.84	0.74	0.86	0.78	1.02	1.19	0.344 ^a^	0.9	0.76	1.04	1.62	0.514 ^a^
Days/week	1.66	1.54	1.69	1.51	1.85	1.62	0.566 ^a^	1.81	1.5	1.52	1.77	0.447 ^a^
**Obstetric data**												
Gestational age							0.663 ^b^					0.546 ^b^
28 to 37 weeks	7	5.6%	4	4.9%	3	4.8%		7	6.1%	0	0	
38 to 41 weeks	111	88.8%	55	67.9%	56	90.3%		90	78.3%	21	75%	
42 weeks or more	7	5.6%	3	3.7%	4	6.5%		6	5.2%	1	3.6%	
Type of delivery							0.685 ^b^					0.924 ^b^
Vaginal delivery	57	45.6%	27	33.3%	30	48.4%		45	39.1%	12	42.9%	
Instrumental vaginal delivery	9	7.2%	6	7.4%	3	4.8%		8	7%	1	3.6%	
Induced vaginal delivery	24	19.2%	10	12.3%	14	22.6%		19	16.5%	5	17.9%	
Instrumental and induced vaginal delivery	7	5.6%	3	3.7%	4	6.5%		7	6.1%	0	0	
Scheduled cesarean	13	10.4%	7	8.6%	6	9.7%		11	9.6%	2	7.1%	
Emergency cesarean	15	12%	9	11.1%	6	9.7%		13	11.3%	2	7.1%	
Complications												
Episiotomy (yes (n.%))	17	13.7%	8	9.9%	9	14.5%	0.434 ^b^	15	13%	2	7.1%	0.453 ^b^
Tear (yes (n.%))	43	34.7%	21	25.9%	22	35.5%	0.441 ^b^	35	30.4%	8	28.6%	0.515 ^b^
Postpartum hemorrhage (yes (n.%))	28	22.6%	15	18.5%	13	21%	0.554 ^b^	23	20%	5	17.9%	0.444 ^b^
Parity												
Primiparous (n.%)	70	56%	33	40.7%	37	59.7%	0.712 ^b^	60	52.2%	10	35.7%	0.358 ^b^
Multiparous (n.%)	55	44%	29	35.8%	26	41.9%		43	37.4%	12	42.9%	
**Number of children**	1.19	0.77	1.15	0.74	1.23	0.79	0.576 ^a^	1.16	0.758	1.32	0.84	0.381 ^a^
**Latest birth data**												
Birth weight (kg)	3.25	0.52	3.26	0.57	3.24	0.47	0.833 ^a^	3.22	0.53	3.39	0.44	0.155 ^a^
Birth length (cm)	49.66	4.13	49.13	4.9	50.19	3.13	0.150 ^a^	49.46	4.43	50.59	2.08	0.246 ^a^
**ODI score**	2.99	4.12	5.68	4.12	0 ^c^	0 ^c^	<0.001 ^a^	3.31	4.12	1.72	3.89	0.028 ^a^
**PSQI score**	11.54	6.82	9.71	4.15	13.57	8.46	<0.001 ^a^	8.52	1.87	23.41	6.2	<0.001 ^a^
**EPDS score**	13.62	7.14	15.5	5.15	11.54	8.38	<0.001 ^a^	16.97	2.7	0.46	2.01	<0.001 ^a^
**TSK score**	8.42	2.7	7.47	2.84	9.47	2.09	<0.001 ^a^	7.9	2.52	10.44	2.45	<0.001 ^a^

PLPP—postpartum lumbopelvic pain; PPD—postpartum depression; SD—standard deviation; kg—kilograms; cm—centimeters; ODI—Oswestry Disability Index; PSQI—Pittsburgh Sleep Quality Index; EPDS—Edinburgh Postnatal Depression Scale; TSK—Tampa Kinesiophobia Scale. The level of significance was set at *p* < 0.05. (^a^) The difference between groups was determined according to the *p*-value based on Student’s t-test. (^b^) Proportions were analyzed using the Chi-square test. (^c^) An ODI score of 0 in women without PLPP indicates the absence of disability, consistent with the scoring structure of the Oswestry Disability Index.

## Data Availability

The data presented in this study are available from the corresponding author upon request. The data are not publicly available due to privacy and ethical restrictions.
